# DNA Methylation of Heparanase Promoter Influences Its Expression and Associated with the Progression of Human Breast Cancer

**DOI:** 10.1371/journal.pone.0092190

**Published:** 2014-03-14

**Authors:** Fei Jiao, Shi-yu Bai, Ying Ma, Zhong-hai Yan, Zhen Yue, Yuan Yu, Xin Wang, Juan Wang

**Affiliations:** 1 Department of Biochemistry and Molecular Biology, Binzhou Medical College, Yantai, Shandong Province, People's Republic of China; 2 Department of Clinical Laboratory, Tai'an City Central Hospital, Tai'an, Shandong Province, People's Republic of China; 3 Department of Medicine, Columbia University, New York, New York, United States of America; 4 Department of Clinical Laboratory, the PLA 107th Hospital Affiliated to Binzhou Medical College Yantai, Shandong Province, Jinan Military Area Command, People's Republic of China; 5 Department of Biotechnology, Binzhou Medical College, Yantai, Shandong Province, People's Republic of China; University of Navarra, Spain

## Abstract

Heparanase promotes tumor invasion and metastasis in several malignancies including breast cancer. However, the roles and regulation mechanisms of heparanase during breast cancer progression are still not fully understood. The aim of this study is to determine the differential regulation of heparanase gene expression in specific stages of breast cancer by DNA methylation. We detected levels of heparanase expression and DNA methylation patterns of its promoter in breast cancer cell lines (MCF-7 and MDA-MB-435) and clinical tissues, respectively. It has been observed that heparanase is highly expressed in the invasive MDA-MB-435 cells with low methylation modification in the heparanase promoter. In contrast, lower expression of heparanase in MCF-7 cells is accompanied by higher methylation in the promoter. Treatment of MCF-7 cells with 5-aza-2′-deoxycytidine (5-aza-dC), a potent demethylating agent, results in induction of heparanase expression and higher invasion potential *in vitro* and leads to an advantage of tumor formation *in vivo*. In 54 tissue samples, cancer samples at late stages (stage IV) showed the highest heparanase expression accomplished by little DNA methylation. On the contrary, methylation prevalence is highest in normal tissue and inversely correlated with heparanase expression. A significant correlation between DNA methylation and clinical stage was demonstrated (*p* = 0.012). Collectively, these results demonstrate that DNA methylation play the regulation role in heparanase gene in different stages of breast cancer and present a direct effect on tumor progression.

## Introduction

The mammalian heparanase is the only endo-β-D-glucuronidase responsible for the degradation of heparan sulfate (HS) chains from heparan sulfate proteoglycans (HSPGs), which is the main polysaccharide component of the extracellular matrix (ECM) and basement membrane (BM) [1]–[2]. Taking into account the fact that degradation of ECM and BM comprises an initial and essential step for cancer cells to invade and metastasize, it is plausible that overexpression of heparanase may facilitate many aspects of tumor development, including migration, invasion and metastasis. Indeed, a large number of studies have clearly linked heparanase expression with the process of tumorigenesis and invasion in a wide number of malignancies, including breast cancer [3]–[4].

Breast cancer is the most common malignant disease affecting women of all age group globally. Despite of recent improvements, the mortality rate of breast cancer is still as high as around 20% at 5 years, in which most patients died from subsequent metastasis often occurring in several preferential sites including bone, lung, liver and brain [5]. Heparanase is closely associated with breast tumorigenesis, invasion and metastasis. A number of studies showed that overexpression of heparanase mRNA and protein was observed both in the *in situ* and invasive components of ductal and lobular origins [6]. In breast carcinoma cell lines, its abundance and enzymatic activity correlate with the aggressiveness. Similarly, the heparanase are preferentially overexpressed in human breast tumors when compared with the normal counterpart [7]. The correlation between heparanase expression and estrogen receptor (ERs) levels confirmed by tissue array further signified its clinical relevance [8]. For *in vitro* systems, cell models demonstrated that silencing heparanase in breast cancer cells could decrease their invasion and adhesion [9]. It is also the case in xenograft animals that overexpression of heparanase in low-metastatic tumor cells confers a highly invasive phenotype [10]. Although a significant correlation of heparanase overexpression is coupled with the progression of breast cancer, the underlying mechanisms remain unclear.

Aberrant patterns of DNA methylation in cancers are commonly observed, with a global hypomethylation of whole genome accompanied by region-specific hypermethylation [11]–[12]. Because cancer progression requires many changes in the normal program of gene expression, it stands to reason that aberrations in DNA methylation play a critical role in the changes in gene expression involved in cancer progression and metastasis [13]. Recently, methylation of heparanase promoter has also been involved in its expression regulation in cancer cell lines [14]. However, previous works also indicated that not all tumor cells expressed heparanase, which indicate that there are different regulatory mechanisms of heparanase expression among tumors. Besides, although heparanase expression and its function in tumor invasion have been well studied, little is known about the epigenetic mechanism that governing the expression of heparanase transcription in breast cancer with different potentials of invasion and metastasis.

To investigate whether DNA methylation is associated with the regulation of heparanase expression during breast cancer progression, we performed detailed methylation analysis by methylation-specific PCR (MSP) combined with pyrosequencing in breast cell lines and clinical samples with different invasion capacity. First, the methylation patterns of the 5′-regulatory region of heparanase gene were evaluated in MCF-7 and MDA-MB-435 cells, two cell lines representing the early and the late stages of the disease, respectively. Further, we determined the effect of 5-aza-2′-deoxycytidine (5-aza-dC), an inhibitor of DNA methyltransferase, on heparanase expression and invasion capacity of both cell lines *in vitro*. The effect of drug treatment on tumorigenesis of MCF-7 was further assessed by a xenograft model *in vivo*. Finally, clinical specimens with different invasive stage were tested to identify whether there is a correlation between DNA hypomethylation and heparanase overexpression during breast cancer progression.

## Materials and Methods

### Ethics Statement

Access to patient samples and anonymous analysis of data was approved by the Institutional Review Board for Human Research at Binzhou Medical College (BZMC-JLF-201012). Written informed consents were obtained from all participants, and all procedures were approved by the ethics board of Binzhou Medical College. All animal experiments were approved by the Binzhou Medical College Animal Care Committee. Animal care protocols conducted were in accordance with animal care committee guidelines to minimize pain and discomfort to animals.

### Cell Lines and experiment design

MCF-7 and MDA-MB-435 cells were obtained from the Shanghai Institute of Biochemistry and Cell Biology, Shanghai, China. Cells were maintained in Dulbecco's Modified Eagle's medium (DMEM) containing glucose at 4.5 g/L supplemented with 10% fetal calf serum (FCS), L-glutamine and 1% penicillin-streptomycin (Invitrogen Gibco, USA). The cells were incubated under a humidified atmosphere of 5% CO_2_ at 37°C. In the 5-aza-dC treatment group, MCF-7 and MDA-MB-435 cells were treated for 7 days with 5-aza-dC (Sigma Aldrich, Ontario, Canada) at final concentrations of 0.5 µM, 5 µM and 10 µM in the regular medium. The medium in the wells was replaced with fresh medium twice a week for both control and treatment groups by maintaining the desired concentration of the drug and subcultured when the cells were about 80–90% confluent.

### Tissue samples

All tissue samples were collected from surgical specimens of patients who underwent a mastectomy at the Affiliated Hospital of Binzhou Medical Colledge, Binzhou, China. A total of 44 patients with infiltrating ductal carcinoma were enrolled. The tumor-node-metastasis (TNM) stages were determined using the official classification method (Stage I: n = 26; Stage IV: n = 18) [15]. These patients did not receive chemotherapy or radiotherapy before surgical operation. Ten normal breast tissues obtained from surgically removed tissues from patience with fibroadenoma as control.

### Methylation-specific PCR (MSP)

Genomic DNA from cells and tissues was extracted with the DNeasy Tissue Kit (Qiagen Inc., Valencia, CA) according to the manufacturer's instructions. After genomic DNA quantification, 1 µg of genomic DNA underwent bisulfite modification utilizing the EZ DNA Methylation-Direct Kit (Zymo Research, Orange, CA, USA). The bisulfite-converted DNA was resuspended in Tris-EDTA (TE) buffer and stored at −80°C until the samples were ready for analysis.

Modified DNA was amplified in a total volume of 20 µl solution containing 1×PCR buffer, 1.5 mM MgCl_2_, 0.2 µM of each primer, 200 µM of each dNTP and 1 U Platinum Taq Polymerase (Life Technologies, Carlsbad, CA). Primers were designed to detect the methylation status of CpG sites using the MethPrimer program (http://itsa.ucsf.edu/~urolab/methprimer) [16]. Primer sequences for unmethylated PCR (MSP-U) and methylated reaction (MSP-M) were listed as in Table 1. Final products were electrophoresed for 20 min at 100 V in a 2% agarose gel. A single band in unmethylated PCR product indicated both alleles of heparanase gene were unmethylated. The presence of a product only in the methylated reaction indicated both alleles of heparanase gene were methylated. Samples that were positive for both methylated and unmethylated reactions were classified as having partially methylated heparanase alleles. Both complete methylation and partial methylation are defined as positive for a given sample.

**Table 1:** Primer sets used for RT-PCR, Real-time RT-PCR, MSP and pyrosequencing.

Primer set	Primers	Sequence	Product size (bp)	Application
HPA	Forward Reverse	5′- ATCTTTGGCCTAAATGCGTTAT -3′ 5′- CATCTTAGCCGTCTTTCTTCG -3′	282	RT-PCR
	Forward Reverse	5′-GAATGGCCCTACCAGGAGCA-3′ 5′-TCCAGTCCTGAGCAGTTTGC-3′	122	Realtime RT-PCR
GAPDH	Forward Reverse	5′- AAGGTCGGAGTCAACGGATTT -3′ 5′- AGATGATGACCCTTTTGGCTC -3′	351	RT-PCR
	Forward Reverse	5′-ACCACAGTCCATGCCATCAC-3′ 5′-AGTAGAGGCAGGGATGATGT-3′	108	Realtime RT-PCR
HPA/MSP	Forward Reverse	5′- TATTCGAGGGTTAGAGGGATATTC -3′ 5′- GAAAATAACCGAAATCCAAACG -3′	193	MSP-M
	Forward Reverse	5′- TTTATTTGAGGGTTAGAGGGATATTT -3′ 5′- ACAAAAATAACCAAAATCCAAACAC -3′	197	MSP-U
HPA/Pyro	Forward Reverse Sequencing	5′- TTTATTAGAGGGTTAGAGGGATATT -3′ 5′- Biotin-AACATCCCCAACCAAACTTCCT -3′ 5′-GGGATATTAGGAGTTATTAGAATGG-3′	119	pyrosequencing

Abbreviations: HPA: heparanase; GAPDH: glyceraldehyde-3-phosphate dehydrogenase; MSP: methylation-specific PCR; MSP-U: amplicons for unmethylated MSP; MSP-M: amplicons for methylated MSP; Pyro: pyrosequencing.

### Characterization of heparanase methylation by pyrosequencing

To quantify the pattern of DNA methylation in heparanase promoter, pyrosequencing was performed using bisulfite converted DNA from cell lines or tissues above mentioned. Bisulfate treated DNA was then eluted in 20 µl volume and 1 µl of it was used for PCR amplification. PCR was performed with one of the PCR primers biotinylated to allow purification of single-stranded DNA templates. The reaction contained the following components: 3.0 mM MgCl_2_, 200 µM dNTPs, 0.2 µM primers, 1 U Platinum Taq Polymerase (Life Technologies, Carlsbad, CA) and 10 ng of bisulfite-converted DNA per 50 µl reaction. PCR cycling conditions were: 94°C×15 min; 45 cycles of 94°C×30 s, 50°C×30 s and 72°C×30 s; and final extension of 72°C×5 min. Following purification, 20 µl PCR products were sequenced by the Pyrosequencing PSQ96 HS System (PSQ H96A, Qiagen Pyrosequencing, Valencia, CA, USA). The sequencing primers were designed by using PyroMark Assay design software ver. 2.0.1.15 (Qiagen, Valencia, CA, USA). The assay was designed to evaluate the methylation status of 7 CpG sites in one sequencing reaction. The primer sequences are described in Table 1. The methylation status of each locus was analyzed individually as a T/C SNP using Pyro Q-CpG Software (Qiagen Pyrosequencing), which converts pyrograms to numerical values for peak heights and calculates the proportion of methylation at each base as a C/T ratio. Then, the average methylation rate of heparanase in a given sample across these 7 CpG sites was obtained.

### RNA extraction, semi-quantitative RT-PCR and Real-time RT-PCR

Total cellular RNAs from MCF-7 and MDA-MB-435 with/without drug treatment groups and tissue RNA were extracted using TRIzol (Invitrogen) according to the manufacturer's protocol. The primers used for semi-quantitative reverse transcription–polymerase chain reaction (RT-PCR) and Real-time RT-PCR were designed based on the heparanase mRNA sequence (GenBank accession number: AF144325) and listed in Table 1. Human glyceraldehyde-3-phosphate dehydrogenase (GAPDH, GenBank accession number: BC083511) was served as internal control. For semi-quantitative RT-PCR, the cDNA was amplified in a PCR machine (Eppendorf) under the following conditions: 95°C×5 min; 30 cycles of 95°C×30 s, 55°C×30 s and 72°C×30 s; and final extension of 72°C×5 min. The PCR products were separated by 1.5% agarose gel electrophoresis and stained with ethidium bromide. Real-time RT-PCR with QuantiTect SYBRGreen Kit (Qiagen) was performed using Rotor-Gene RG3000 PCR cycler (Corbett Research): An initial denaturation at 95°C for 5 min, followed by 35 cycles of 95°C for 30 s, 55°C annealing for 20 s, and extension at 72°C for 20 s. Fluorescence was detected at 585 nm at each extension step of 72°C. Relative expression levels were normalized to the expression of GAPDH mRNA and calculated by the 2^−ΔΔCt^ method.

### Western blots

MCF-7 and MDA-MB-435 with/without drug treatment were lysed in lysis buffer (10 mM HEPES, 142.5 mM KCl, 5 mM MgCl_2_, 1 mM EDTA, 0.2% Nonidet P-40, 0.1% aprotinin, and 1 mM phenylmethylsulfonyl fluoride (PMSF), pH 7.2) at 4°C for 30 min, and the lysates were centrifuged (12,000 g) at 4°C for 15 min. Total protein (25 µg) was subjected to 10% sodium dodecyl sulfate-polyacrylamide gel electrophoresis (SDS-PAGE) and then electro-transferred to PVDF membranes (Millipore, Boston, USA). After being blocked with 5% fat-free milk in Tris-buffered saline Tween-20 (pH 7.6), the PVDF membranes were incubated with primary antibodies against heparanase (1∶500, Santa Cruz Biotechnology, Santa Cruz, USA) and GAPDH (1∶1000, Santa Cruz Biotechnology, Santa Cruz, CA) overnight at 4°C, followed by HRP-conjugated secondary antibody. Immunoreactive bands were detected using the Chemilucent ECL detection system (Millipore). Optical band density was quantified by the Image J software (version 1.43, NIH, Bethesda, USA).

### Analysis of Cell cycle distribution

Cell cycle distribution was monitored by flow cytometry analysis. Briefly, Untreated MCF-7 and MDA-MB-435 cells were used as negative controls. Cells were treated with 5-aza-dC alone or in combination with 1 µM OGT2115, a heparanase inhibitor (Tocris Bioscience, Bristol, UK), respectively [17]. Cells with or without treatment were trypsinized, washed with phosphate-buffered saline (PBS) and fixed with 75% ethanol overnight at −20°C. The cells were washed twice with 1×PBS and added 1 ml of propidium iodide (PI, Sigma, USA) staining solution (50 µg/ml) to cell pellet. Then, 50 µl of RNase A stock solution was added and incubated for 3 h at 4°C. The cell cycle was analyzed by a flow cytometry (Beckman Coulter, Inc., USA).

### Boyden chamber Matrigel invasion assay

Cell invasion assays were performed as previously described [18]. Briefly, Untreated MCF-7 and MDA-MB-435 cells were used as negative controls. Cells were treated with 5-aza-dC alone or in combination with 1 µM OGT2115, respectively. BD Matrigel Basement Membrane Matrix (BD BioScience, concentration approx. 9 mg/ml) was mixed 1∶1 with PBS was allowed to polymerize in transwell inserts (Corning) for at least 1 hour at 37°C. Total 5×10^5^ cells with/without treatment in 0.1 ml of serum-free medium were plated in the upper chamber containing the Matrigel-coated membranes. Serum-containing media acted as chemo-attractants in the lower chambers. After incubation for 48 h, the invaded cells at the bottom of the membrane were stained with 0.1% crystal violet and counted under a light microscope with a ×400 magnification. Ten randomly selected fields were examined and the average number of cells invaded was calculated.

### *In vivo* tumorigenicity assay

Twelve female BALB/c nude mice, 5–6 weeks old, were obtained from the Institute of Zoology, Chinese Academy of Sciences (Beijing, China)., and were randomly divided into four groups: control (untreated, n = 3), 0.5 µM (n = 3), 5 µM (n = 3) and 10 µM (n = 3). Before injection, MCF-7 cells were maintained in the regular medium containing 5-aza-dC with the desired concentration for 72 h. On day 4, 100 µl of single cell suspensions (2.0×10^7^ cells/ml) from untreated and treated groups were subcutaneously inoculated into lower back of nude mice. One week before inoculation, a 60-d release estrogen pellet (0.72 mg β-estradiol, Innovative Research of America) was implanted subcutaneously in each mouse. Tumor growth was evaluated by measuring the maximum diameter (A) and the minimum diameter (B) of tumor mass with a caliper at day 0, day 6, day 12, day 18, day 24 and day 30, respectively. The mean tumor volumes were calculated according to the formula V = A×B^2^/2. At the end of the study, mice were sacrificed by cervical dislocation and tumor masses were removed and weighed.

### Immunohistochemistry

Clinical samples were fixed for 24 h at 4°C in 4% formaldehyde, dehydrated and embedded in paraffin, sectioned (thickness, 5 µm) for immunohistochemical analysis of heparanase. Briefly, after deparaffinization and rehydratation, slides were washed and incubated with 2.5% H_2_O_2_ for 30 min to quench endogenous peroxide activities and then were blocked with 1% bovine serum albumin in PBS for 1 h at room temperature. A monoclonal antibody against heparanase (1∶500; Santa Cruz Biotechnologies, Santa Cruz, CA) was used as the primary antibody for detecting protein expression. Immunodetection was performed by incubation with a specific biotinylated secondary antibody followed by use of the Vectastain ABC kit (Vector Laboratories, Burlingame, CA). 3,3′-Diaminobenzidine (Vector Laboratories, Burlingame, CA) was used as the developing reagent followed by a hematoxylin counterstain. Slides were examined under a light microscope (Olympus, Tokyo, Japan) with a ×200 magnification.

### Data analysis

The chi-square test was used to analyze differences in the rate of each variable. A two-tailed Student *t*-test and an analysis of variance (ANOVA) were used to detect differences in the mean values of the variables. A value of *p* less than 0.05 is considered statistically significant. All statistical analyses were performed using SPSS 15.0 software package (Chicago, IL, USA).

## Results

### Methylation status of heparanase promoter and the effect of 5-aza-dC treatment in breast cancer cell lines

**Figure 1:**
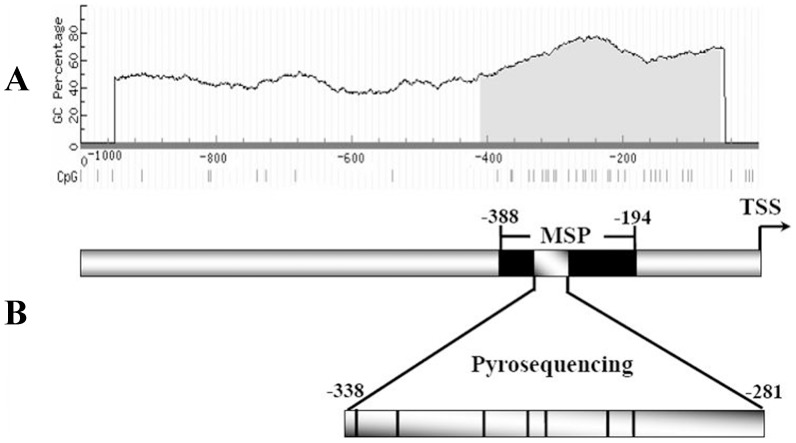
CpG analysis and amplification regions of human heparanase gene. (A) Modified output of MethPrimer program (Li and Dahiya, 2002). Coordinates are given in relation to the transcription start site (TSS); A 355 bp CpG island (−55–−411, grey region) is evident in the upstream of the gene. Vertical lines indicate relative positions of CpG dinucleotides. (B) Schematic representation of amplification regions for Methylation-specific PCR (MSP) and pyrosequencing. Black box indicated amplicon for MSP located at −194–−388 relative to TSS. Region from −281–−338 including 7 CpG dinucleotides (vertical lines in the grey box) was performed pyrosequencing reaction. Primer sequences and expected PCR product sizes are shown in Table 1.

**Figure 2:**
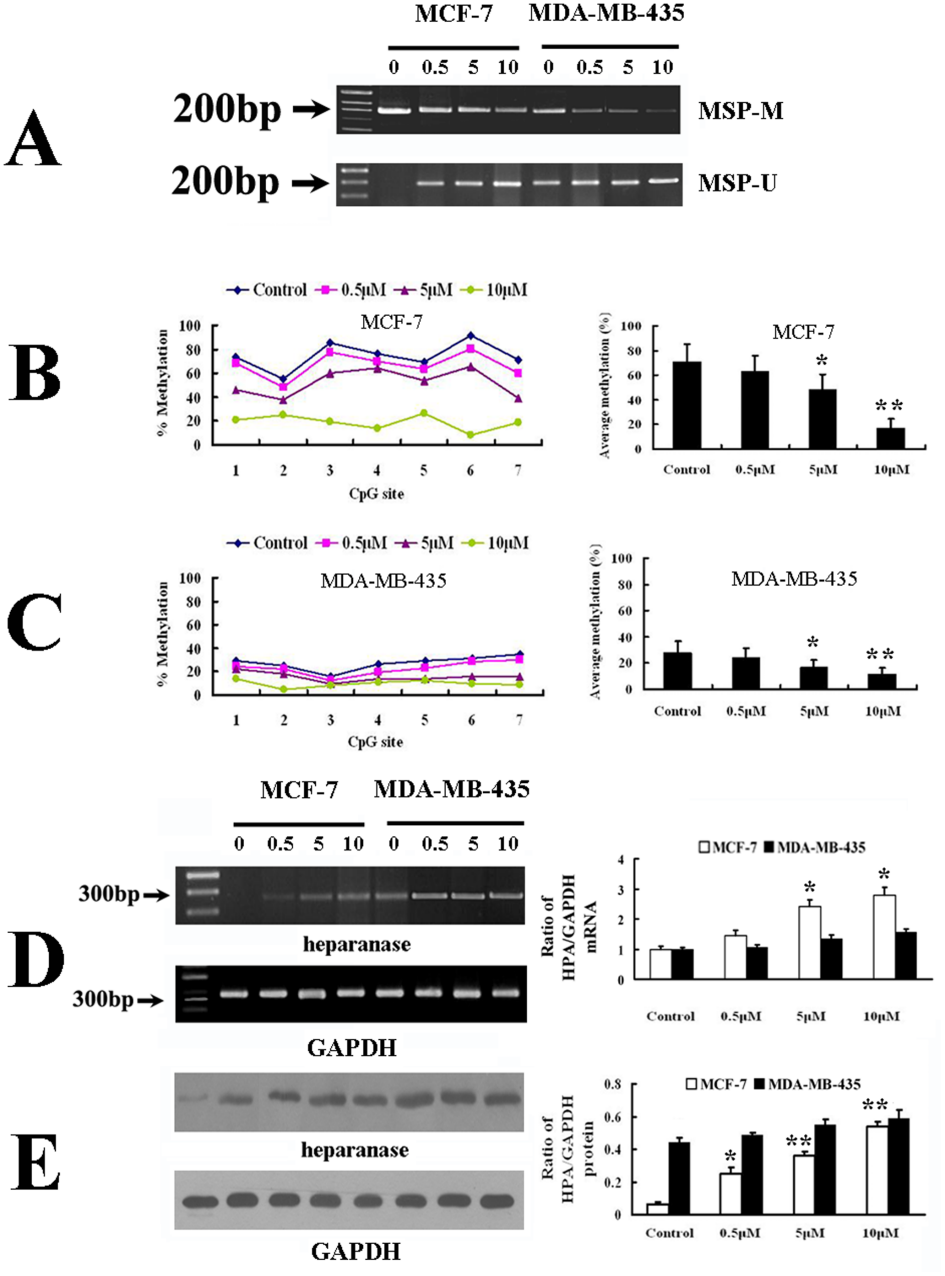
Effects of 5-aza-dC on the DNA methylation and expression of heparanase in MCF-7 and MDA-MB-435 cells. (A) MSP analyses showed that all the three different concentrations of 5-aza-dC (0.5 µM, 5 µM and 10 µM) can attenuate the DNA methylation of heparanase in a dose-dependent manner, especially in MCF-7. The untreated cells served as a control. (B and C) Pyrosequencing results of DNA methylation changes in MCF-7 and MDA-MB-435 cells with 5-aza-dC treatment. Quantitative analysis showed that DNA methylation of heparanase decreased significantly in a dose-dependent manner, especially in MCF-7. (D) Analyses of mRNA abundances in MCF-7 and MDA-MB-435 cells treated with different concentrations of 5-aza-dC (0.5 µM, 5 µM and 10 µM) by semi-quantitative RT-PCR (left panel) and Real-time RT-PCR (right panel). (E) Western blot detections of of heparanase expression in MCF-7 and MDA-MB-435 cells treated with different concentrations of 5-aza-dC (0.5 µM, 5 µM and 10 µM). (*p<0.05, **p<0.01 compared with the control.). Abbreviations: MSP: methylation-specific PCR; MSP-U: amplicons for unmethylated MSP; MSP-M: amplicons for methylated MSP.

We first detected CpG methylation status of heparanase promoter in two breast cell lines with and without 5-aza-dC treatment using MSP and pyrosequencing, respectively. The schematic of CpG island in heparanase promoter and regions analyzed by MSP and pyrosequencing were illustrated in Figure 1A and Figure 1B, respectively. In MSP, two pairs of PCR primers were designed to span 11 CpG sites within the heparanase promoter region. Without 5-aza-dC treatment, the MSP detection showed that methylated CpG sites in heparanase promoter were higher in MCF-7 cells than those in MDA-MB-435 cells. After treated with gradient concentrations of 5-aza-dC, the intensity of bands amplified by methylated primers (MSP-M) was significantly decreased in a dose-dependent manner, especially in MCF-7 (Fig. 2A). Subsequently, a 58-bp region located from −281 to −338 in heparanase promoter containing 7 CpG sites was analyzed by pyrosequencing. In groups of MCF-7 cells without and with 5-aza-dC treatment, the sequencing results showed that CpGs methylation percentage were 74.6%, 64.9%, 52.5% and 19.0%, respectively (Fig. 2B). For MDA-MB-435 cells, the rates were 27.6%, 23.1%, 15.8% and 10.0%, respectively (Fig. 2C). These results indicated that 5-aza-dC treatment can inhibit DNA methylation obviously, especially in MCF-7 cells.

### 5-aza-dC treatment exhibit variant effect on the expression of heparanase in MCF-7 and MDA-MB-435 cells

To further determine whether DNA methylation/demethylation played a role in heparanase expression, we tested the effect of 5-aza-dC treatment on the expression of heparanase in MCF-7 and MDA-MB-435 cells. Both conventional and Real-time PCR demonstrated that the expression level of heparanase mRNA was elevated paralleling with the alteration in methylation of heparanase before and after 5-aza-dC treatment in MCF-7, which have relatively low levels of heparanase mRNA before drug treatment (Fig. 2D). For MDA-MB-435 cells, however, only a slight increase was observed in response to 5-aza-dC treatment (Fig. 2D). Similarly, increased expression of heparanase protein was also only in MCF-7 cells (Fig. 2E).

### 5-aza-dC treatment inhibit cell proliferation of breast cancer cells

**Figure 3:**
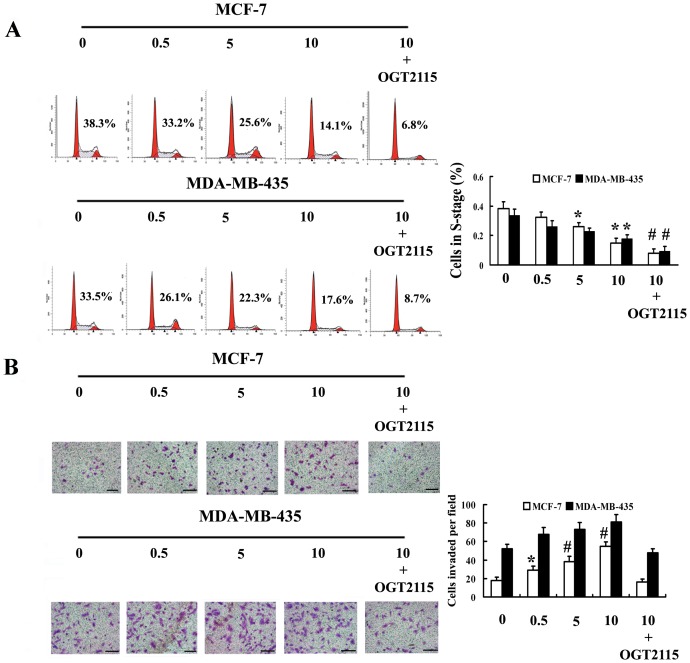
Assay of cell cycle and invasion potential for MCF-7 and MDA-MB-435 cells after 5-aza-dC treatment. (A) Cell cycle distributions of MCF-7 and MDA-MB-435 cells treated with different concentrations of 5-aza-dC (0.5 µM, 5 µM and 10 µM). The effect of heparanase on the distribution of cell cycle was further investigated by the exposure of the cells to OGT2115 (1 µM). The percentage of S-phage cells among groups was given in each peak plot of flow cytometry detection. (B) Matrigel assay for MCF-7 and MDA-MB-435 cells after 5-aza-dC treatment. Specificity of heparanase in changing the cell invasive capacity was further confirmed by co-incubation of 5-aza-dC-treated cells with OGT2115 (1 µM). Cells that penetrated the polycarbonate membrane were stained with 0.1% crystal violet and examined by light microscopy (×200). The mean number of cells per field on the lower surface of the filter was determined from ten random microscopic fields per filter and from three filters per cell type. The untreated cells served as a control. (*p<0.05, #*p*<0.01 compared with the control.). Scale bar = 100 µm.

To investigate whether methylation inhibitors were relevant to regulating cell proliferation, flow cytometry analysis was performed to detect the changes of cell cycle distribution after 5-aza-dC treatment. Our results demonstrated that the percentage of detected cells in S phage decreased significantly in 5-aza-dC-treated groups compared to those in control (*p*<0.05; Fig. 3A). Incubation of these cells with OGT2115, an inhibitor of heparanase, can increase the inhibitory effect of 5-aza-dC on cell proliferation (*p*<0.01; Fig. 3A). This observation indicated that, at least in part, heparanase is involved in the changes of proliferation in tumor cell treatment with 5-aza-dC. In addition, the antiproliferative effect of drugs seems to be more significant in MCF-7 than that in MDA-MB-435 cells. This observation suggested that the antineoplastic role of 5-aza-dC is relevant to regulating cell cycle, most likely due to an inhibition of S phase.

### Demethylating agent increase the invasive capacity of MCF-7 cells

We next carried out Matrigel invasion assay to examine the effect of heparanase expression changes resulting from methylation alteration on the invasive capacity of these cells. In control groups that no drugs were applied, MDA-MB-435 cells expressing high levels of heparanase are able to invade through the Matrigel, whereas only few MCF-7 cells are able to penetrate the polycarbonate membrane. In contrast, following 5-aza-dC treatment, the low-invasive nature of MCF-7 cells was reversed based on the observation that a significantly increased number of tumor cells invaded through the Matrigel (Fig. 3B, upper panel). For MDA-MB-435 cells, the increase of invasion potential is not significant after drug application (Fig. 3B, lower panel). To further confirm that heparanase plays key role in enhancing cell invasion, OGT2115 was applied to inhibit heparanase activity. Significant decrease of invasive capacity was observed after incubation of cells with OGT2115, implying that the increase in tumor cell invasion after treatment with 5-aza-dC is due to the induction of heparanase expression (Fig. 3B).

### Inhibition of DNA methylation of heparanase in MCF-7 facilitate its tumorigenesis *in vivo*

**Figure 4:**
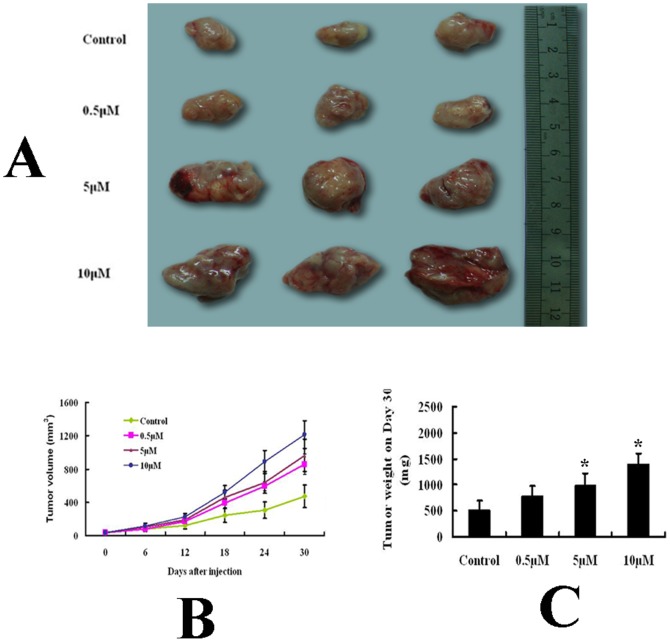
Role of 5-aza-dC in the tumorigenicity of MCF-7 cells *in vivo*. Hypodermic injection of drug-treated MCF-7 cells in nude mice established subcutaneous xenograft tumors. On day 30 of the experiment, mice (n = 3) from each group were sacrificed and their tumors were dissected and weighed. (A) Gross appearance of tumors on day 30. (B) Time-course measurements of tumor volume in different groups. (C) Analysis of tumor weights in different groups on day 30. Tumor weights in drug-treated groups were significantly larger than those in control groups. (*p<0.05 compared with the control.).

Regarding that the effect of drug treatment on heparanase expression of MDA-MB-435 cells is not significantly *in vitro* both at mRNA and protein level, only MCF-7 cells were selected for the tumor formation assay *in vivo*. Fig. 4A showed the tumors collected from animals at the end of experiments. The average volume of tumor mass in the control group was significantly smaller than those in the treatment groups (472±133 mm^3^ vs 859±121 mm^3^, 964±193 mm^3^ and 1215±165 mm^3^, respectively, Fig. 4B). Similar results of tumor growth *in vivo* were observed at day 6, 12, 18 and 24, respectively. The tumor mass weights at day 30 among groups were shown in Fig. 4C. In the control and treated (0.5 µM, 5 µM and 10 µM) groups, tumors weight were 518±172 mg, 788±190 mg, 986±387 mg and 1402±331 mg, respectively (Fig. 4C). The results indicated that demethylating agents treatment of MCF-7 facilitate its tumorigenicity in a dose-dependent manner.

### Methylation profile of the heparanase gene in clinical samples

**Figure 5:**
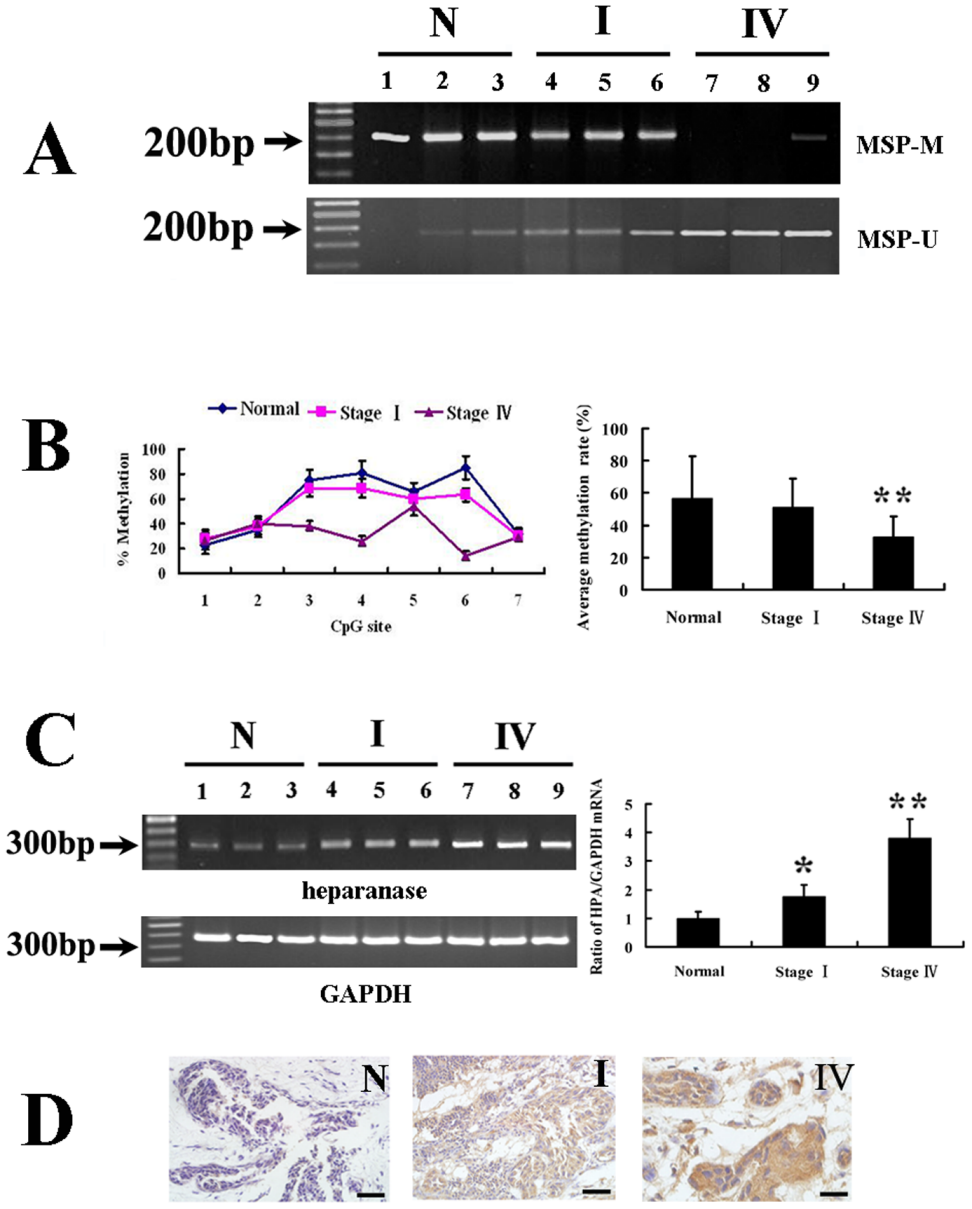
DNA methylation status and expression of heparanase in breast cancer tissues. (A) Representative results of MSP in breast cancer tissues. (B) Pyrosequencing results of DNA methylation changes in breast cancer tissues. Quantitative analyses of DNA methylation in heparanase among different groups were showed in the histogram. (C) Analyses of mRNA expression in breast tissues by semi-quantitative RT-PCR (left panel) and Real-time RT-PCR (right panel). (D) Representative immune-staining of heparanase in normal control and cancer tissues (×200). Staining of heparanase in control tissue is very weak. In contrast, the strongest expression of heparanase was observed in cancer tissues at stage IV. Scale bar = 100 µm (*p<0.05, **p<0.01 compared with the normal control.). Abbreviations: MSP-U: amplicons for unmethylated MSP; MSP-M: amplicons for methylated MSP; N: normal tissue; I: cancer tissue (Stage I); IV: cancer tissue (Stage IV).

To determine the DNA methylation status of heparanase in three different groups of breast samples, methylation patterns were examined by MSP and pyrosequencing, respectively. Among three groups, the differences of mean age were not significant (*p* = 0.470) (Table 2). By MSP detection, the methylation of heparanase was identified in 41.3% of late breast cancers (Stage IV), 80.8% of early breast cancers (Stage I) and 90.0% of control samples (Table 2). Representative results of MSP were shown in Fig. 5A, indicating a decrease of DNA methylation during cancer progression. For pyrosequencing, the plotting diagram demonstrated that methylation of detected CpG sites fluctuated significantly (Fig. 5B). Overall, methylation level of heparanase promoter is lower in stages IV tumors than those of stages I tumors and normal tissues (32.5%±12.9%, 50.9%±18.2% and 56.6%±25.9%, respectively, Fig. 5B and Table 2). These results implied that DNA methylation of heparanase gene can alter dynamically during breast cancer progression.

**Table 2:** The methylation patterns of the heparanase gene in control and breast cancer with different stage.

	Control (n = 10)	Stage I (n = 26)	Stage IV (n = 18)	*p*-value
Age (years)	42.3±11.6	45.4±10.2	47.8±12.7	0.470^a^
heparanase methylation cases (%)	9(90.0)	21(80.8)	7(41.3)	0.004^b^
Mean methylation rates (%)^c^	56.6±25.9	50.9±18.2	32.5±12.9	0.001^a^

a ANOVA.

b Chi-square test.

c The average methylation rate in a given sample was calculated from 7 CpG sites detected by pyrosequencing.

### Evaluation of heparanase expression in tissues by RT-PCR and immunohistochemical staining

Despite that heparanase mRNA can be detected by semi-quantitative RT-PCR in nearly all samples, the intensity of products were elevated in tumors (Stage IV) with low-methylated gene (Fig. 5C, left panel). These observations were further confirmed by Real-time RT-PCR (Fig. 5C, right panel). The results indicated that heparanase mRNA levels are inversely correlated with the methylation status of its promoter. Representative results of RT-PCR among groups were shown in Fig. 5C. Immunohistochemistry analyses provided similar results in protein level (Fig. 5D).

### Correlations between DNA methylation of heparanase and clinicopathological features in breast cancer

In breast cancer samples, the correlations between DNA methylation of heparanase and several clinicopathological parameters were further investigated (Table 3). Methylation status of heparanase was classified into two groups according to the results of MSP. The evaluated clinicopathological parameters included tumor size, status of node metastasis, clinical stage, estrogen and progesterone receptors (ER and PgR), and human epidermal growth factor receptor 2 (HER2). A correlation between heparanase methylation and clinical stage was observed (*p* = 0.012). In contrast, the methylation of heparanase had no apparent associations with the following prognostic factors: tumor size (*p* = 0.078), node metastasis (*p* = 0.133), ER positivity (*p* = 0.101), PgR positivity (*p* = 0.528), and HER2 expression (*p* = 0.196).

**Table 3:** Correlation of clinicopathological factors with the methylation status of the heparanase gene in breast cancer samples.

	Patients with methylated heparanase (%) (n = 28)	Patients with unmethylated heparanase (%) (n = 16)	*p*-value
Tumor size Mean± SD* (cm)	2.27±0.47	2.56±0.52	0.078^a^
Node metastasis			0.133^b^
Positive	5 (17.9)	7(43.8)	
Negative	23(82.1)	9(56.2)	
Clinical Stage			0.012^b^
I	21(75.0)	5(31.3)	
IV	7(25.0)	11(68.7)	
Estrogen receptor			0.101^b^
Positive	19(67.9)	6(37.5)	
Negative	9(32.1)	10(62.5)	
Progestogen receptor			0.528^b^
Positive	17(60.7)	12(75.0)	
Negative	11(39.3)	4(25.0)	
HER2			0.196^b^
Positive	16(57.1)	13(81.3)	
Negative	12(42.9)	3(18.7)	

* standard deviation.

a Student *t*-test.

b Chi-square test.

## Discussion

It is well established that breast cancer is the most common cancer and the leading cancer-related cause of death among women worldwide. New and better combinations of treatments such as chemotherapy, hormonal therapy, and radiotherapy as well as earlier detection through mammography screening programs have contributed to the improved prognosis. The role of heparanase in sustaining the pathology of malignant tumors was confirmed by a variety of reports [19]–[21]. Both over-expression and silencing of the heparanase gene clearly indicated the involvement of heparanase in tumor invasion, metastasis and angiogenesis [22]. The preferential overexpression of heparanase has also been demonstrated in breast cancer [23]. These studies suggest that heparanase may be served as a molecular target for cancer therapy.

Meanwhile, studies about heparanase regulation showed that its expression is involved in multifaceted mechanisms [24]. In breast malignancies, a causal role for demethylation in cancer metastasis is supported by the fact that treatment of non-metastatic breast cancer cells with demethylation agents increases their invasiveness, and that treatment of invasive breast cancer with agents that reverse demethylation results in inhibition of invasion and metastasis [25]–[26]. The association between DNA hypomethylation and tumor size and histological grade for tumors further provides evidence for its importance in the prognosis of patients [27].

However, mechanisms responsible for heparanase induction are incompletely understood. In the present study, we tested whether DNA methylation is involved in the differential regulation of heparanase during breast cancer progression. We first detected the expression level and methylation of CpG sites within the heparanase promoter in MCF-7 and MDA-MB-435 cells, which represents early stage human breast cancer with low invasive capacity and late stage breast cancer with high metastatic capacity, respectively. Elevated heparanase levels have been observed in MDA-MB-435 cells in our study. Meanwhile, methylation of promoter in heparanase is much higher in MCF-7 than that in MDA-MB-435 cells, indicating that there is an inverse correlation between heparanase abundance and its promoter methylation. The result is quite consistent with previous studies regarding the regulation of heparanase by promoter methylation in prostate and bladder cancers [28]–[29].

The treatment of DNA methyltransferase inhibitors, such as 5-aza-dC, to evaluate the DNA methylation on selected genes has been widely accepted, and it can induce the re-expression of tumor suppressor genes by demethylating promoter CpG sites [30]–[31]. In this study, after treatment with 5′-azaC for 7 days, an induction of heparanase mRNA expression and protein abundance in MCF-7 cells resulted in an increase in invasive capacity of these cells. For MDA-MB-435 cells, however, the reactivation of heparanase genes by DNA demethylation is not as significant as in MCF-7 cells. These observations are plausible taking into account that untreated MDA-MB-435 cells possess relatively low level of DNA methylation compared with MCF-7 cells. Based on this consideration, we suppose this different result may cause by cell line-specific and dose-depend performance of 5-aza-dC. These results are reasonable regarding the different status of DNA methylation in both cell lines and clearly demonstrate the tight association between the drugs induced hypomethylation and restoration of heparanase activity at cellular levels.

As it is reported that heparanase expression is correlated with tumor size and clinical progression stage [12], it is interesting to speculate that heparanase may be participating in tumor growth *in vivo*. Here, using breast cancer cell xenograft model, we observed that BALB/c nude mice subcutaneously injected with MCF-7 cells treated with 5-aza-dC resulted in larger tumor volume and presented greater in tumor mass weight than those in the untreated control group. While consistent with several previous studies [28], [32], the observations in the present study are opposite to the findings obtained by Ateeq et al. [5]. With the fact in mind that sequential changes of gene expression during treatment of demethylation agents, the discrepancy may partly result from the time of drug treatment [33]–[34]. Besides, the different sites of inoculation may influence the tumor growth *in vivo*, because a series of publications indicated that the behavior of cancer cells in animal models was altered in response to the varying microenvironment resulting from incubation sites [35]–[36]. Further investigations should be warranted to elucidate potential mechanisms using tumors from xerografts.

It should been noted that, in many instances, changes of DNA methylation in cultured cells *in vitro* are different from clinical tissues. Therefore, it is worthy of performing further investigations at clinical level using samples from patients. Generally consistent with available investigations, in normal tissues, only little mRNA and very limited protein of heparanase were detected accomplished by significant CpG methylation [37]. In contrast, the expression of heparanase is predominant in cancer samples with higher grades, whereas cytosines of CpG in these tumor tissues were almost low-methylated. Therefore, the data show that there is a negative correlation between methylation and protein level of heparanase which is in line with previous reports [38]–[39]. When we use TFSEARCH database (http://www.cbrc.jb/research/db/TFSEARCH) to analyze these biased methylated CpG sites for consensus binding sites of the known transcription factors with the threshold score above 90.0, many transcription binding factors including GATA-1, c-Ets and USF were predicted (data not shown). These transcription binding factors are proved previously that can couple with heparanase promoter and play an important role in tumor invasion and metastasis by modulating the remodeling of ECM [40]–[41]. Thus, it is reasonable to imply that the methylation regulation mechanism of heparanase may interfere with the binding of these transcription factors, leading to the heparanase expression in different carcinoma stages. Further study using proper methods, such as chromatin immunoprecipitation assay (CHIP), to confirm these potential transcription factors specific binding to heparanase promoter in breast carcinoma is worth to be warranted.

In this study, we also revealed heparanase was more frequently methylated in breast cancer samples at early stage than in those at late stage. In contrast, no correlation was found between heparanase methylation and prognostic factors, such as tumor size, node metastasis, ER/PgR positivity and HER2. However, it should be mentioned that the correlation between heparanase expression and its status of DNA methylation is still inconclusive for clinical samples. Several possible explanations may be contributed to this situation. First, DNA methylation outside the detected region of heparanase promoter might be involved, and our MSP and pyrosequencing study only spans very limited region in promoter [42]–[43]. As global epigenetic alterations in cancer, it can be expected that the contribution of DNA methylation alternations in single gene is limited. DNA methylation detections in more candidate genes and more CpG sites will make the correlations more accurate and convincing [44]. Secondly, the limited population examined and therefore the high heterogeneity among breast cancer patients in current study might lead to unexpected bias. Thirdly, the regulation of heparanase is involved many factors in addition to cytosine methylation, including transcriptional and post-transcriptional regulation or other epigenetic mechanisms (e.g. histone modification, small non-coding RNA, environmental exposure induced methylation *et al*) [45]–[47].

Another important issue should be noted is the true origin of the human MDA-MB-435 cell line. Because some melanocyte-related genes can be expressed in MDA-MB-435 cells, this lead to the speculation that MDA-MB-435 cells might be melanoma rather than breast in origin, implying that its use as a model of human breast cancer is unsuitable [48]–[49]. However, recent findings about transdifferentiation or lineage infidelity in cancers may provide new insights into this issue. It has been demonstrated that aberrant co-expression of multi-lineage markers via transdifferentiation or lineage infidelity can occur frequently in breast cancer, not only in cell lines but also in freshly resected specimens [50]–[53]. Therefore, it may not be exclusively reliable to determine the tissue origin of a cancer cell line based on molecular signatures derived from gene expression profiling [54].

In summary, this study points to epigenetic control of heparanase expression and cancer phenotype in cell lines, animal models and clinical samples of human via site-specific DNA methylation. Our results provide convincing evidence for cytosine methylation as a molecular mechanism involved in transcriptional regulation of heparanase gene expression and demonstrate that DNA methylation of heparanase do undergo distinct changes during breast tumor progression.
